# Evaluation of a Language Translation App in an Undergraduate Medical Communication Course: Proof-of-Concept and Usability Study

**DOI:** 10.2196/31559

**Published:** 2021-12-02

**Authors:** Anne Herrmann-Werner, Teresa Loda, Stephan Zipfel, Martin Holderried, Friederike Holderried, Rebecca Erschens

**Affiliations:** 1 Department of Internal Medicine VI/Psychosomatic Medicine and Psychotherapy University Hospital Tuebingen Tuebingen Germany; 2 Competence Centre for University Teaching in Medicine, Baden-Wuerttemberg Faculty of Medicine Eberhard-Karls University Tuebingen Germany; 3 Deanery of Students’ Affairs Faculty of Medicine Eberhard-Karls University Tuebingen Germany; 4 Department of Strategic Medical Development and Quality Management University of Tuebingen Tuebingen Germany; 5 Institute for Healthcare and Public Management University of Hohenheim Stuttgart Germany; 6 eHealth Research-Group Department of General, Visceral, and Transplant Surgery University of Tuebingen Tuebingen Germany

**Keywords:** undergraduate medical students, translation app, simulation, physician-patient communication, mHealth, mobile applications, digital health, app development, language translation, translation apps

## Abstract

**Background:**

Language barriers in medical encounters pose risks for interactions with patients, their care, and their outcomes. Because human translators, the gold standard for mitigating language barriers, can be cost- and time-intensive, mechanical alternatives such as language translation apps (LTA) have gained in popularity. However, adequate training for physicians in using LTAs remains elusive.

**Objective:**

A proof-of-concept pilot study was designed to evaluate the use of a speech-to-speech LTA in a specific simulated physician-patient situation, particularly its perceived usability, helpfulness, and meaningfulness, and to assess the teaching unit overall.

**Methods:**

Students engaged in a 90-min simulation with a standardized patient (SP) and the LTA iTranslate Converse. Thereafter, they rated the LTA with six items—*helpful*, *intuitive*, *informative*, *accurate*, *recommendable*, and *applicable—*on a 7-point Likert scale ranging from 1 (*don’t agree at all*) to 7 (*completely agree*) and could provide free-text responses for four items: general impression of the LTA, the LTA’s benefits, the LTA’s risks, and suggestions for improvement. Students also assessed the teaching unit on a 6-point scale from 1 (*excellent*) to 6 (*insufficient*). Data were evaluated quantitatively with mean (SD) values and qualitatively in thematic content analysis.

**Results:**

Of 111 students in the course, 76 (68.5%) participated (59.2% women, age 20.7 years, SD 3.3 years). Values for the LTA’s being *helpful* (mean 3.45, SD 1.79), *recommendable* (mean 3.33, SD 1.65) and *applicable* (mean 3.57, SD 1.85) were centered around the average of 3.5. The items *intuitive* (mean 4.57, SD 1.74) and *informative* (mean 4.53, SD 1.95) were above average. The only below-average item concerned its *accuracy* (mean 2.38, SD 1.36). Students rated the teaching unit as being excellent (mean 1.2, SD 0.54) but wanted practical training with an SP plus a simulated human translator first. Free-text responses revealed several concerns about translation errors that could jeopardize diagnostic decisions. Students feared that patient-physician communication mediated by the LTA could decrease empathy and raised concerns regarding data protection and technical reliability. Nevertheless, they appreciated the LTA’s cost-effectiveness and usefulness as the best option when the gold standard is unavailable. They also reported wanting more medical-specific vocabulary and images to convey all information necessary for medical communication.

**Conclusions:**

This study revealed the feasibility of using a speech-to-speech LTA in an undergraduate medical course. Although human translators remain the gold standard, LTAs could be valuable alternatives. Students appreciated the simulated teaching and recognized the LTA’s potential benefits and risks for use in real-world clinical settings. To optimize patients’ and health care professionals’ experiences with LTAs, future investigations should examine specific design options for training interventions and consider the legal aspects of human-machine interaction in health care settings.

## Introduction

Communication with patients ranks among the most important tasks for physicians and is thus an integral aspect of medical training [1-3]. Many institutional bodies and national catalogues of learning objectives have even designated communication with patients as a core competency [4-8]. However, several circumstances (eg, reduced consciousness and high emotionality) can impair communication with patients [9,10]; of them, language barriers can especially put timely, sufficient medical care at risk [11,12]. In nonmedical contexts, internet- and app-based digital translation services have become widely used to overcome such language barriers.

Albeit to a lesser extent than in the general public, the use of such translation services, particularly language translation apps (LTA), has gained traction in medical settings. Owing to increased globalization, migration, and refugee resettlement during the 21st century, patients often cannot speak the language spoken where they receive medical treatment and may thus be at risk of receiving less effective health care [13-15]. In response, human translators have been shown to benefit health care delivery in numerous ways; hence, various attempts have been made to train medical students or physicians to act as translators [16-20]. Although human translators are currently the gold standard for obtaining information from patients, obtaining their informed consent, and delivering negative news to them [10,21,22], such services may not always be available owing to timing and financial limitations. In such cases, digital technology such as LTAs seem to offer the second-best option [10,23,24]. LTAs generally function in one of three ways: text to text (ie, translation of a word or sentence from text into new text), text to speech (ie, translation of text from a tappable dictionary into voice output), and speech to speech (ie, translation of spoken sentences into voice output) [25].

In any case, LTAs are doubtlessly preferable to ad hoc alternatives such as relying on relatives, who may be too emotionally involved and thus prone to potentially fatal translation errors, or staff members who speak the same language as the patient, which would violate patient confidentiality and data security and could precipitate misunderstandings due to a lack of clinical and medical knowledge [21,26-29]. Nonetheless, guidelines applicable to communication via human translators may also be relevant when using LTAs, including ensuring direct communication, maintaining eye contact, talking to the person instead of the device, and using simple, clear, and sufficiently audible language [30,31].

In emergency medicine, studies have shown that using LTAs can overcome language barriers [32,33]. In particular, participants in those studies reported greater satisfaction with the more domain-specific app QuickSpeak than the generic Google Translate, although in both cases, they were worried about the inaccuracy of the translations [10,34-40]. Other studies have involved investigating the use of LTAs in clinical settings and shown their usefulness in simple communicational situations [34,41,42].

LTAs designed for clinical application often require the use of simple sentences. As a case in point, by using a text-to-speech app that simplified open-ended questions into closed-ended ones, Narang et al [33] found good user satisfaction and improvements in communication with patients with limited English proficiency. At the same time, inaccuracy in machine translation has been documented in various LTAs [40,42-44] and could precipitate misdiagnoses, incorrect prescriptions, and general mistreatment [45,46]. In sum, using LTAs in physician-patient communication demands caution, and physicians need to be trained in the adequate professional use of such apps [34,47].

Against that background, we conducted a proof-of-concept pilot study to examine the use of a speech-to-speech LTA in an undergraduate medical course, particularly its perceived usability, helpfulness, and meaningfulness in a simulated specific physician-patient situation, and to assess the teaching unit overall. Because the setting was simulated, we did not account for legal aspects (eg, data security) that would apply in clinical settings.

## Methods

### Setting and Participants

The proof-of-concept study was conducted in the Medical Faculty at the University of Tuebingen, Germany, between April and June 2019 in a medical communication course designed for the second preclinical year of medical school. Whereas participation in the course was mandatory, participation in the study was voluntarily, and 111 medical students were invited to participate. All participating students gave their written informed consent to participate, and data were collected anonymously.

### Teaching Unit and Study Procedure

The teaching unit in this study was a 90-min seminar within the medical communication course taught by experienced instructors at the Department of Psychosomatic Medicine and Psychotherapy. All students had previously attended a lecture on the general aspects of physician-patient communication and how to inquire into and document a patient’s medical history. They had also completed a repeat session on specifically assessing the history of present illness (HPI) in which they had practiced with a standardized patient (SP) presenting with chronic back pain.

The seminar on the LTA began with an introductory lecture on handling language-nonconcordant patients. Students also received information on how to work with human translators, including common pitfalls to avoid, and organizational background information specific to Tuebingen University Hospital (eg, how to request and pay human translators). Afterward, during the encounter with the SP, one medical student per 10-student group acted as the attending physician. The interaction was followed by a feedback session on general communication strategies and the overall management of the situation. The session ended with an interactive discussion on the usage of an LTA in physician-patient communication and appropriate medical strategies in the management of this patient case.

### Standardized Patient

The SP was a 20-year-old male from Syria who spoke Arabic, a foreign language chosen owing to its relative frequency among patients in German hospitals and the low probability that participating students would understand or speak it. The SP was a young traveler who had experienced acute-onset nausea and vertigo hours before, which had worsened when he presented at the emergency department at 3 AM. Although the patient could not speak German or English, the attending physician’s task was nevertheless to obtain some basic information about the patient and his HPI using the LTA. Full instructions are provided in Textboxes 1 and 2.

**Instruction for students acting as the attending physician.**

Setting:

Emergency department, 3 AM.

You are the attending physician on your 4th night shift this week.

Case:

A 20-year-old male presents with acute-onset vertigo and nausea. The highly experienced on-duty nurse tells you, with slight exasperation, that the patient can communicate in Arabic only and that all attempts to gather basic information thus far have been futile. More important, the patient is in obvious distress (e.g. restlessly turning on the stretcher and clutching a kidney dish), and time seems of the essence. Knowing that no other staff on the ward can translate Arabic, you consider the option to request a professional translator. However, you are also aware that procuring a translator won’t be easy at 3 a.m. As an alternative, you remember that one of your colleagues had introduced you to a language translation app, and you decide that now is the time to try it. After all, what do you have to lose?

Task:

Take the patient’s HPI using the app on the iPhone. You have 10 min.

**Instruction for the standardized patients.**

Setting:

Emergency department, 3 AM.

You are _______________ (insert name), a 20-year-old from Syria who has been travelling across Europe with a friend for several weeks. Although you do not speak any German or English, you have managed quite well thus far.

This evening, you experienced a sudden onset of nausea and vertigo. You haven’t been drinking alcohol or taken any drugs. The vertigo is rotational, similar to being on a merry-go-round, not a sailboat, and you feel the constant urge to vomit, even though you have not vomited thus far. Although lying on your back initially helped, your posture no longer affects your symptoms, and turning your head rapidly especially worsens your vertigo. You have never experienced a comparable condition, and you are unaware of any family history of vertigo.

You are usually an open-minded, easy-going person who loves to travel. You are in Europe for the first time, and so far, you have had lots of fun and appreciated all of the impressions made and opportunities encountered on your journey. Currently, however, you feel rather unwell and slightly scared because you can’t judge the seriousness of your situation, and it doesn’t help that you don’t understand what people are saying. On the plus side, you very much like the young doctor taking care of you. You appreciate their effort to communicate with you on an app and thus try your best to communicate given the circumstances.

Remember that you speak Arabic exclusively. Only respond to whatever the app translates for you, even if you know that the original question in German was somewhat different. Please use simple sentences and only respond to what you’ve been asked (e.g. don’t add information).

If you’re asked any question not listed in these instructions, then please improvise. Remember, the session is part of a medical communication course in the second year of medical school. The simulation does not focus on the medical content as much as the general communication techniques and the specific situation of communicating via the app.

The encounter will last approximately 10 min.

### LTA: iTranslate Converse

The app used, iTranslate Converse available for Android, Windows, and Mac, was chosen for its benefits identified by Khander et al [48] that we considered important for our simulation—that is, a wide range of available languages, ease of navigation and a high score (2.5/2.7) for “application comprehensiveness.” It has also been shown to produce translations of similar quality to that of human translators, at least with simple sentences [35]. Preliminary tests for usability were also conducted by 2 authors (AHW and SZ).

The LTA was downloaded to an iPhone 7 device from the faculty’s IT Department; the phone was not connected to the hospital’s Wi-Fi, had no SIM card but had its languages preset to German and Arabic. The app was downloaded using Wi-Fi, accessed with the Apple ID of one author (AHW), and the connection was terminated immediately afterward because the LTA can be run offline.

Before students commenced the SP encounter, they were allotted time to become familiar with how the LTA worked. To translate speech, the student, either as the attending physician or patient, had to tap and hold a button while speaking, and releasing the button generated an audio translation. The system recognized the language spoken and automatically switched between the 2 preset languages.

### Questionnaire

We developed a questionnaire with reference to the literature, models (eg, Unified Theory of Acceptance and Use of Technology) and ratings by expert panels [10,49-51]. Before the first seminar, the questionnaire had undergone cognitive pretesting by using the so-called “think aloud” method, in which the respondent concurrently verbalizes thoughts when responding to questionnaire items [52,53]. Consequently, minor adaptions to the questionnaire were made, and it was administered after the teaching unit but before the interactive discussion. The questionnaire collected demographic information (ie, age and gender) and ratings of the use of the LTA, the latter with 6 items on a 7-point Likert scale ranging from 1 (*don’t agree at all*) to 7 (*completely agree*). The 6 items were (1) *helpful* (ie, able to support the task), (2) *intuitive* (ie, easy to use), (3) *informative* (ie, able to gather all necessary information), (4) *accurate* (ie, able to provide correct translations), (5) *recommendable* (ie, advisable for use by patients and clinical staff), and (6) *applicable* (ie, likely to be employed for personal use). Following those items, complementary free-text responses were requested for four additional items: (7) general impression of the LTA, (8) the LTA’s benefits, (9) the LTA’s risks, and (10) suggestions for improvement. These questions were added to obtain a deeper insight into students’ considerations.

### Teaching Unit Evaluation

Students anonymously evaluated the teaching unit on a secure platform for teaching assessment used by the faculty members for all courses at the university’s medical school. The grading system used in German schools (1=*excellent*, 6=*insufficient*) was employed.

### Respondents and Nonrespondents

At the beginning of the study, a questionnaire was placed on each medical student’s desk. Students who answered and submitted the questionnaire were considered respondents, whereas those who left the questionnaire unanswered or did not submit it were considered nonrespondents.

### Statistical Analysis

A sample size calculation was conducted with a 95% CI, population proportion of 50%, and a population size of 120, which resulted in a sample size of 92 respondents. The data were evaluated both quantitatively and qualitatively. Quantitative statistical analyses were performed with SPSS for Windows (version 25.0) under the assumption that the variables followed a normal distribution. First, for reliability analysis, the Cronbach α for internal consistency was computed to assess the 6 items in the quantitative part of the questionnaire (ie, Items 1-6). The internal consistency was satisfactory (α=.86), and reliability could not be improved by deleting items [54]. Corrected item–total correlations for all 6 items ranged between .45 and .81, and mean (SD) values were calculated. The final 4 items addressed in free-text responses (ie, Items 7-10) were evaluated in thematic content analysis using Microsoft Excel as coding software [55]. Themes in the data set were identified, analyzed, and documented. During content analysis, the reviewers familiarized themselves with the data and developed codes. After themes were sought, examined, and specified, results of the analysis were interpreted.

### Ethics

Ethics approval for the study was obtained by the local ethics committee (No. 443/2018BO2).

### Data Availability Statement

Full data are available on reasonable request by the corresponding author.

## Results

### Demographic Information

Of the 111 students in the course, 76 (68.5%) participated in the study. Most were women (n=45, 59.2%), and all were from 17 to 40 years of age (mean 20.66 years, SD 3.26 years).

### Rating of the LTA

The mean rating across the first 6 items (ie, items 1-6) was only slightly above average (mean 3.64, SD 1.36). For the individual items, ratings for *helpful*, *recommendable*, and *applicable* were average. Students rated the LTA’s being *intuitive* and *informative* as slightly above average (mean 4.52, SD 1.95) but its *accuracy* as rather below average (mean 2.38, SD 1.36). Table 1 reports the individual ratings of the 6 dimensions.

**Table 1:** Items regarding features of the language translation app.

Dimension	Rating (1=*don’t agree at all*, 7=*completely agree*), mean (SD)
(1) Helpful	3.45 (1.79)
(2) Intuitive	4.57 (1.74)
(3) Informative	4.52 (1.95)
(4) Accurate	2.38 (1.36)
(5) Recommendable	3.33 (1.65)
(6) Applicable	3.57 (1.85)

### Analysis of Free-Text Responses

Regarding items 6-10, most general impressions regarding the LTA contained largely critical comments about its accuracy. The students noticed, especially following comments from the SP and bilingual classmates, the possibility of severe translation errors, especially in translations from Arabic to German. Students also reported that the LTA largely failed to compute long, complex, or open-ended questions, and students instead suggested using close-ended questions to “get to the point.” Many students reported worrying that planning and administering misguided follow-up or unnecessary interventions owing to linguistic misunderstandings could harm patients. Students additionally raised concerns about the technical challenges that LTAs can present (eg, poor connectivity or updates).

Regarding the LTA’s benefits, students considered the app very useful for emergency situations and other brief conversations. Beyond that, they envisioned using the LTA more in hospital contexts than in ambulatory ones. A particularly positive aspect mentioned was that the LTA allows creating transcripts of dialogues, albeit only in its paid upgraded version. Another advantage was the LTA’s cost-effectiveness relative to human translators and its potential use in translating uncommon languages and dialects not always known by hospitals’ human translators.

Concerning the LTA’s risks, students emphasized not only concerns about inaccuracy and its consequences but also the risk of fragmented, ineffective physician-patient communication. By using the LTA as an intermediary, many students experienced increased distance between themselves and the SP and added that the LTA needlessly prolonged the task of taking the SP’s medical history. Students also reported worrying about losing empathy for patients and their symptoms by using the LTA. In particular, to assess mental distress or psychological comorbidities, they expressed doubts that the LTA would transmit the interpersonal information correctly. Furthermore, students were concerned that they would accidentally make offensive or politically incorrect statements to patients owing to the LTA’s mistranslation. Other feedback focused on the extent to which the LTA guaranteed data confidentiality and whether machines such as LTAs would soon replace human interpreters.

Finally, regarding suggestions for improving the LTA, students generally aligned with their risk assessments by expressing a desire for more accurate translations. Considering the context of application, however, they contemplated the usefulness of predefined questions as a means to simplify the taking of medical history. Along similar lines, students wished for specialized terms adapted to the medical context and a “greater and more diverse vocabulary” both to prevent misunderstandings and to plan more precise interventions. Other students proposed adding pictures or predefined snapshots of difficult situations to improve the LTA’s translation accuracy and ease of use. A final suggestion was for the LTA to reproduce the voice of the respective speaker to make taking the medical history more realistic.

### Teaching Unit Evaluation

Analysis of the free-text responses in the evaluation of the teaching unit revealed that students were interested in the topic and generally liked the idea of including an app in the course’s instruction. They also appreciated the possibility of practicing with the LTA with an SP in a controlled environment and receiving feedback from multiple sources afterward. At the same time, they underscored the topic’s lack of connection to other learning content and demanded a better introduction to the topic, including practice with an SP along with a simulated human translator first. On the whole, students quantitatively rated the teaching unit in the official teaching evaluation system as excellent (mean 1.2, SD 0.54).

## Discussion

Our proof-of-concept pilot study was designed to gain insight into the use of an LTA in a simulated setting in undergraduate medical education.

### Principal Findings

Tested as part of an undergraduate medical curriculum, the LTA was perceived by medical students as being generally useful for the task of taking a HPI during acute care. Students appreciated the teaching unit taught in the seminar, even if they had only general interest in the topic and favored using the gold standard of human translators instead, which corroborates with other published findings [56].

When comparing human variants in translation, the role of the translator demands consideration. Ideally, a translator should act as a “conduit” transferring information neutrally from one party to the other [57,58]. However, depending on the circumstances, additional roles—managers, advocates, cultural mediators, or even co-therapists, to name a few—may equally need to be filled [59,60], none of which LTA can. Despite this limitation, it does guarantee the basic function demanded of a translator—pure information exchange—and students should be made aware of its possibilities.

Although generally appreciative of the teaching unit, students complained that the challenge of using LTAs can be better confronted with more training, especially simulated training in communication with the aid of a human translator. Such training could easily be accommodated by the educational approach of spiral curriculum design [61].

Students’ overall satisfaction with the LTA was high, however, as previous findings have also shown [47]. In particular, medical students considered the LTA easy to handle, possibly owing to its user-friendly interface and the fact that the students’ age group is highly familiar with using mobile apps in their day-to-day lives. Nevertheless, the results suggest that students need to be trained in the professional application of LTAs, as recently stressed [47]. Students also acknowledged the potential of acquiring the necessary information with the LTA, information that they could not have obtained without the app, or at least not as rapidly, which confirms a known effect of using LTAs [62]. Even so, the students could readily specify the potential difficulties and pitfalls of using an LTA in real-world practice. In general, students feared that using an LTA to communicate with patients would threaten the physician’s empathy, which is another known phenomenon of the replacement of human translators [63]. They were also concerned that translation errors could result in maltreatment or misdiagnosis, among other dangerous mistakes, that would jeopardize the patient’s health and life. Their concern echoes findings from other research groups [10,27,35,40].

At the same time, our intended meaning of *errors* needs clarification. So-called “noncatastrophic errors” such as incorrect grammar or awkward translations may be tolerable, whereas critical mistranslations may not only cause confusion but also create the potential for serious harm [40,64]. Students need to be aware of such problems and need to be equipped with strategies to minimize them. After all, professional human translators are as liable to commit translation errors that become medical errors [29]. Similarly, an LTA’s disadvantage may be its inflexibility compared to the flexibility that human interaction offers. With a human translator, at least one person can understand both languages and may be able to detect mismatches between speech and reactions and can adapt to cultural differences and communication-related concerns, whereas machines can accomplish neither task. Nevertheless, as Freyne et al [62] have shown, with repeated use of an LTA, health care professionals cultivate confidence in its translation abilities, possibly because they adapt their way of speaking to accommodate the possibilities and limitations of the app’s functionality. To aid that process, some students wished for predefined sentences or images as a means to minimize misunderstandings. On that topic, the choice of Arabic as the SP’s language might have aggravated the problem in our study because especially rare or non-European languages are prone to translation errors [38,65]. Indeed, more specific apps such as Quick Talk have been shown to be more helpful in emergency medicine settings than Google Translate [10]. Additionally, when used with native speakers on both ends, LTAs can usually produce the correct meaning, even if the translation is not completely accurate [10]. An ideal solution might be a mix of preset questions as options supplemented with images and the additional function of free-text entry.

The reluctance to trust the LTA’s accuracy was also reflected by the fact that whereas all ratings for the 6 items correlated with each other, no intercorrelation emerged between the students’ rating of the LTA’s helpfulness and their assessment of its accuracy, which indicates that the students appreciated using an LTA for collecting the medical history of language-nonconcordant patients but were partly deterred by its technical restrictions.

### Limitations

Our study had several limitations. First, it was conducted during only one semester with medical students from only one faculty in Germany. Those constraints upon the sample and the study limit the generalizability of the findings. Second, only one LTA was used in the study, meaning that the findings might not be applicable for other LTAs. Third, we tested only one language, Arabic, chosen as a compromise between a language encountered often enough amongst patients in hospitals in Germany and a language with little risk of being known by many students, which would have jeopardized their learning experience. Because students participated in the study on a voluntary basis, we cannot exclude selection bias; however, given the number of respondents and their age and gender distribution, the sample can be considered to represent the student population at Tuebingen Medical Faculty in general. Finally, the study was designed as a self-report paper-and-pencil survey with quantitative and open-ended questions. Self-report surveys are generally open to bias, and responses to the items were analyzed in accordance with the level of data available. Consequently, there was no need to compute moderator or between-group analyses.

Despite those limitations, we strongly believe that the pilot study offers valuable insight into the use of a speech-to-speech LTA that offers the possibility of speaking freely, in an undergraduate medical curriculum. Those initial data show that such an LTA can be helpful in obtaining the HPIs of patients in simulated acute care settings. It remains unclear whether this app could be reliably integrated into actual patient care where other additional aspects (eg, data protection and legal liability) would have to be considered.

### Comparison With Prior Work

To the best of our knowledge, this study was the first to examine an LTA in undergraduate medical education, which offers the possibility of speaking freely and thus approximates a normal conversation without language barriers. Findings concerning the evaluation of the LTA used were primarily in line with published results. However, they additionally showed that students need training in the use of LTAs, which confirms the recently identified need among physicians to be properly prepared for using LTAs.

### Conclusions

Our proof-of-concept study revealed that using a speech-to-speech LTA in an undergraduate medical class is feasible. Students primarily benefitted from the feedback from multiple sources as part of the simulation, as well as from becoming familiar with the general possibilities and potential drawbacks of using LTAs.

Although human translators remain the gold standard and are preferred by patients and health care professionals, LTAs might pose a valuable alternative to less favorable options (eg, relying on bystanders and family members) or a valuable addition to the off-the-cuff approach because they do not present the obstacles that human translators often do (eg, timing, cost, and inflexibility) [10,21,26,33,56,66]. Students liked the idea of studying the topic as part of their simulated teaching. However, they also recognized the risks of using such an LTA in clinical settings with real patients.

The COVID-19 pandemic has altered health care in diverse ways, including by increasing the acceptability of telemedical health care solutions. Further investigations should examine changes in the usage and acceptability of LTAs and how training interventions can be designed to optimize patients’ and health care professionals’ experiences with LTAs. At the same time, legal concerns (eg, data security) need to be addressed in future LTA training courses because they are essential to consider when LTAs are intended for use in clinical practice. As a next step, we propose the development of a full-scale training course for undergraduate medical students that addresses communication with language-nonconcordant patients, including algorithms and strategies for using LTAs and the gold standard: face-to-face or video-based human translation.

## Abbreviations

HPI: history of present illness
LTA: language translation app
SP: standardized patient
